# Supplementary material for: Principal cell activity induces spine relocation of adult-born interneurons in the olfactory bulb
Source: Nat Commun. 2016 Aug 31;7:12659. doi: 10.1038/ncomms12659 (PMC5013674; doi:10.1038/ncomms12659)
Supplement: Supplementary Figures — 1-4 [file ncomms12659-s1.pdf]

Supplementary figures

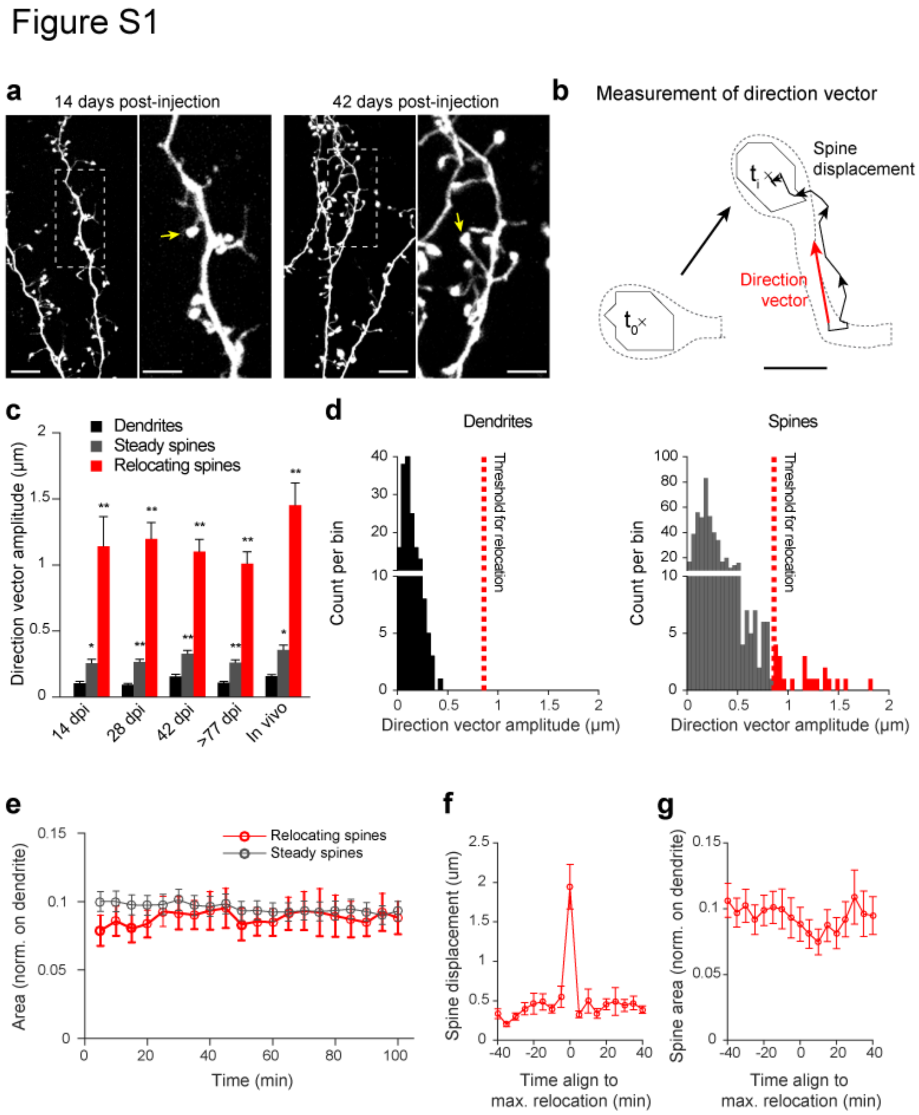

Supplementary Figure 1: Direction vector amplitudes of GC spines and dendrites

a, Location of spines shown in **Fig. 1f.** Scale bars**:** 10 μm for 1st and 3rd panels, and 5 μm for 2nd and 4th panels. **b,** Illustration of the methodology for calculating spine relocation and the average direction vector. The spine head location was calculated for each time-point for 30 to 90 min. Spine relocation was the sum of the distance between positions at each time point normalized to that of the dendrite from which the spine emerged. The direction vector was calculated by averaging the vector between the origin (t = 0 min)

and each time point. The direction vector gave a direct measurement of the directionality of the spine relocation. The greater the amplitude of spine direction, the more the spine moved in a specific direction. Scale bar: 2 µm. **c,** Quantification of direction vector amplitudes for GC dendrites versus spines. n = 25, 34, 31, 28, and 38 dendritic segments and n = 68, 124, 93, 103, and 126 spines from 7, 7, 7, 5, and 16 mice for the 14, 28, 42, 77 dpi, and *in vivo* conditions, respectively. *: $p < 0.01$, and **: $p < 0.001$ compared to dendrite relocation vector calculated with ANOVA with Tukey's post-hoc test. **d,** Histograms comparing the distribution of direction vector amplitudes for 118 dendritic segments (left panel) and 514 randomly selected spines (right panel). The right dashed lines demonstrate the threshold used to classify relocating spines. Note that the threshold is well above the noise level expressed by the distribution of direction vector amplitudes for dendritic segments. **e,** Quantification of volume changes normalized on a local portion of a dendrite for relocating (direction vector > 0.86 µm) and non-relocating (direction vector < 0.4 µm) spines. **f,** Spine displacement between each time point (5-min intervals) aligned to the maximum displacement for the relocating spines shown in **e**. **g,** Volume changes for relocating spines as calculated in **e** aligned to the maximum displacement shown in **f** (n = 13 and 17 dendrites from 10 and 13 cells from 8 and 11 mice for relocating and non-relocating spines, respectively).

Figure S2

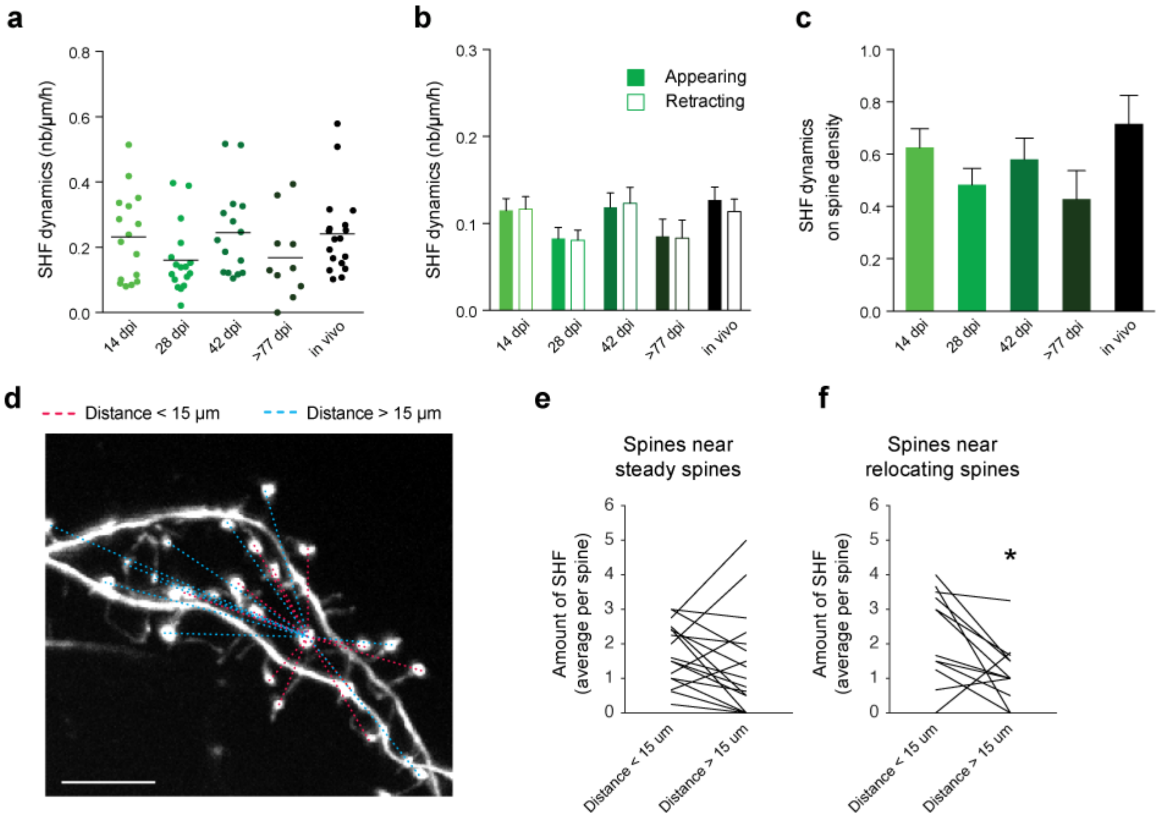

Supplementary Figure 2: SHF dynamic at different maturational stages

a, Quantification of SHF dynamics for different maturational stages of adult-born GC. **b,** SHF dynamics considered separately for appearing and retracting SHF at different maturational stages. **c,** SHF dynamic normalized on spine density for different maturational stages of adult-born GC. No significant differences between groups in **a-c** were observed with a one-way ANOVA with Tukey post-hoc test (n = 16, 17, 15 and 10 cells from 10, 10, 9, and 5 mice for the 14, 28, 42, and 77 dpi conditions, respectively). **d-f,** Analysis of SHF dynamic on the spines located close to and far away from the relocating **(d,f)** and non-relocating **(e)** spines. Scale bar: 10 µm.

Figure suppl. 3

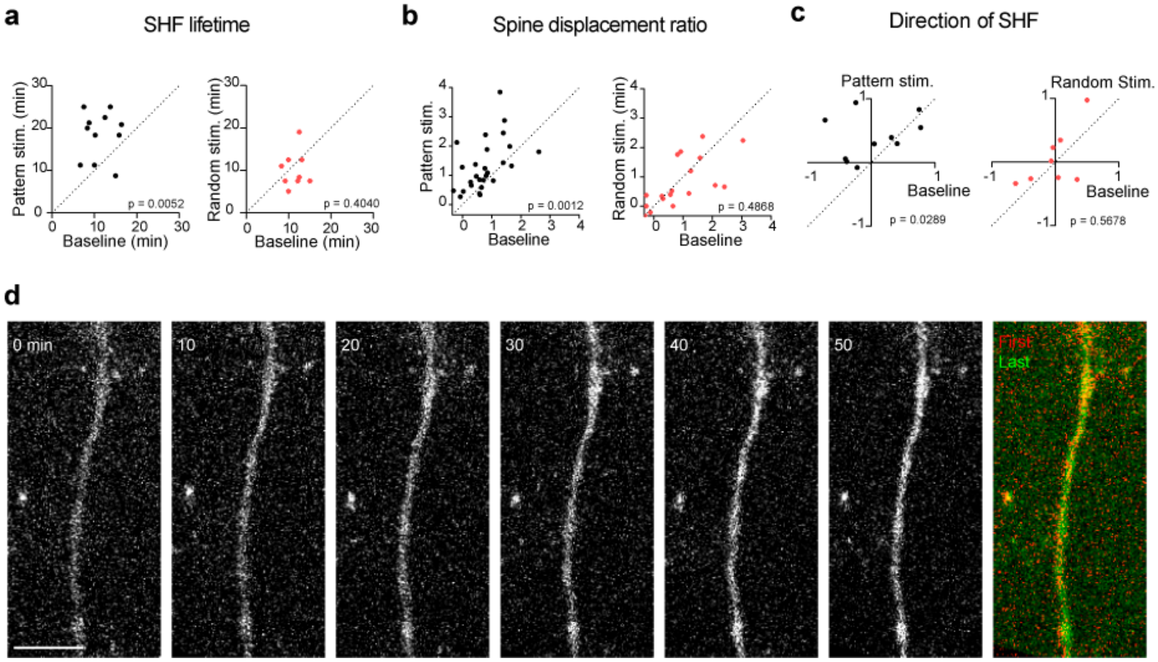

Supplementary Figure 3: Random stimulation pattern of MC does not induce GC spine relocation

a-c, Comparison of physiological (left panel in **a-c**) and random (right panel in **a-c**) patterns of stimulation delivered to a single MC. The random stimulation pattern did not cause an increase in the lifetime of SHF (**a**, right panel), spine direction ratio (**b**, right panel), or direction of SHF growth (**c**, right panel). n = 18 spines from 9 MC-GC pairs from 9 mice and n = 27 spines from 11 MC-GC pairs from 9 mice for the random and physiological stimulations, respectively. p values were calculated using the paired Student's t-test. **d**, Time-lapse imaging of Alexa594-filled MC dendrite showing its stability over the time. Scale bar: 10 µm.

Figure S4

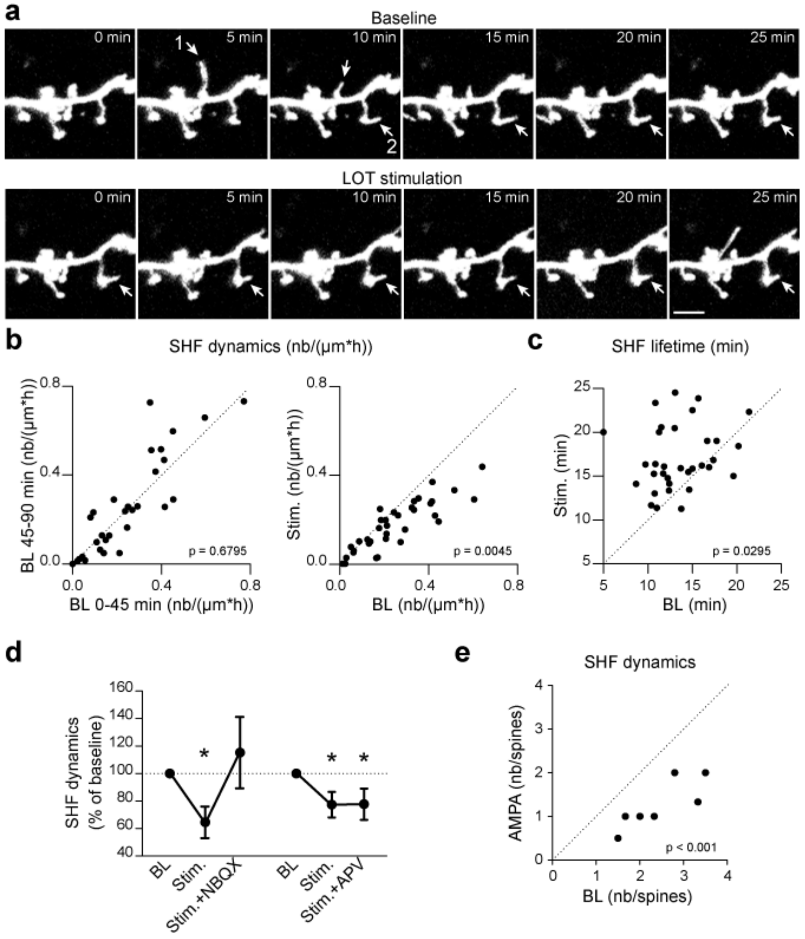

Supplementary Figure 4: Activation of AMPARs is required for SHF stability.

a, Sequence of time-lapse two-photon images of a distal dendrite of an adult-born GC taken at baseline and during lateral olfactory tract (LOT) stimulation. Arrow 1 indicates a spine that did not display SHF dynamics after LOT stimulation. Arrow 2 indicates a SHF that stabilized after LOT stimulation. Scale bar: 5 µm. **b,** Quantification of SHF dynamics of the controls (left panel; n = 30 cells from 12 mice) and during LOT stimulation (right panel; n = 35 cells from 18 mice). **c,** Effect of LOT stimulation on the lifetime of SHF (n = 33 cells from 18 mice). **d,** Effect of the bath application of the AMPA and NMDA

receptor antagonists, NBQX and APV respectively, on the decrease in SHF dynamics observed following MC stimulation. The SHF dynamics were normalized to the baseline value (n = 11 and 12 cells from 7 and 5 mice for NBQX and APV, respectively; *$p < 0.05$ using the Student's paired t-test). **e,** Effect of the local application of AMPA on the SHF dynamics of spines located within 10 μm of the iontophoresis pipette (n = 7 cells from 3 mice).
